# Effect of perioperative sigh ventilation on postoperative hypoxemia and pulmonary complications after on-pump cardiac surgery (E-SIGHT): study protocol for a randomized controlled trial

**DOI:** 10.1186/s13063-024-08416-y

**Published:** 2024-09-04

**Authors:** Zhichang Wang, Qiyu Cheng, Shenglun Huang, Jie Sun, Jingyuan Xu, Jianfeng Xie, Hailong Cao, Fengmei Guo

**Affiliations:** 1 Jiangsu Provincial Key Laboratory of Critical Care Medicine, Department of Critical Care Medicine, Zhongda Hospital, School of Medicine, Southeast University, Nanjing, 210009 China; 2 Department of Anesthesiology, Surgery and Pain Management, Zhongda Hospital, School of Medicine, Southeast University, Nanjing, 210009 China; 3 Department of Cardiothoracic Surgery, Zhongda Hospital, School of Medicine, Southeast University, Nanjing, 210009 China

**Keywords:** Postoperative hypoxemia, Postoperative pulmonary complications, Cardiac surgery, Cardiopulmonary bypass, Sigh, Mechanical ventilation

## Abstract

**Background:**

Postoperative hypoxemia and pulmonary complications remain a frequent event after on-pump cardiac surgery and mostly characterized by pulmonary atelectasis. Surfactant dysfunction or hyposecretion happens prior to atelectasis formation, and sigh represents the strongest stimulus for surfactant secretion. The role of sigh breaths added to conventional lung protective ventilation in reducing postoperative hypoxemia and pulmonary complications among cardiac surgery is unknown.

**Methods:**

The perioperative sigh ventilation in cardiac surgery (E-SIGHT) trial is a single-center, two-arm, randomized controlled trial. In total, 192 patients scheduled for elective cardiac surgery with cardiopulmonary bypass (CPB) and aortic cross-clamp will be randomized into one of the two treatment arms. In the experimental group, besides conventional lung protective ventilation, sigh volumes producing plateau pressures of 35 cmH_2_O (or 40 cmH_2_O for patients with body mass index > 35 kg/m^2^) delivered once every 6 min from intubation to extubation. In the control group, conventional lung protective ventilation without preplanned recruitment maneuvers is used. Lung protective ventilation (LPV) consists of low tidal volumes (6–8 mL/kg of predicted body weight) and positive end-expiratory pressure (PEEP) setting according to low PEEP/FiO_2_ table for acute respiratory distress syndrome (ARDS). The primary endpoint is time-weighted average SpO_2_/FiO_2_ ratio during the initial post-extubation hour. Main secondary endpoint is the severity of postoperative pulmonary complications (PPCs) computed by postoperative day 7.

**Discussion:**

The E-SIGHT trial will be the first randomized controlled trial to evaluate the impact of perioperative sigh ventilation on the postoperative outcomes after on-pump cardiac surgery. The trial will introduce and assess a novel perioperative ventilation approach to mitigate the risk of postoperative hypoxemia and PPCs in patients undergoing cardiac surgery. Also provide the basis for a future larger trial aiming at verifying the impact of sigh ventilation on postoperative pulmonary complications.

**Trial registration:**

ClinicalTrials.gov NCT06248320. Registered on January 30, 2024. Last updated February 26, 2024.

**Supplementary Information:**

The online version contains supplementary material available at 10.1186/s13063-024-08416-y.

## Background

Postoperative pulmonary complications (PPCs) remain a frequent event after on-pump cardiac surgery and mostly characterized by pulmonary atelectasis [1]. Atelectrauma, denoting the cyclic expansion and collapse of atelectatic lung regions, and volutrauma, involving excessive distension of neighboring patent alveoli, are recognized as pivotal contributors to ventilator-induced lung injury (VILI) subsequent to atelectasis [2, 3]. This injurious sequence is associated with hypoxemia, pneumonia, ventilator-induced lung injury, and acute respiratory distress syndrome (ARDS), thereby engendering escalated healthcare resource utilization, delayed patient mobilization, prolonged reliance on supplementary oxygen or mechanical ventilation, and protracted hospitalization [4].

Patients undergoing on-pump cardiac surgery are subject to a convergence of pulmonary insults. The administration of general anesthesia combined with invasive mechanical ventilation initiates a distinctive lung insult recognized as ventilator-induced lung injury [5]. Additionally, cardiac surgery introduces a secondary insult: cardiopulmonary bypass (CPB) elicits a systemic inflammatory response [6], while the application of the aortic cross clamp contributes to pulmonary ischemic injury [7]. Moreover, the frequent necessity for blood transfusions and the experience of postoperative pain significantly contribute to the heightened incidence of postoperative pulmonary complications (PPCs) [8, 9]. Pulmonary atelectasis is very common in this context.

The implementation of targeted perioperative ventilatory strategies aimed at mitigating pulmonary atelectasis and postoperative pulmonary complications (PPCs) represents a well-established approach [10–12]. Numerous investigations have described the concept of lung protective ventilation. Inspired by the results obtained in critical care medicine in patients with acute respiratory distress syndrome (ARDS), the use of low tidal volumes (6–8 mL/kg predicted body weight) has spread to the operation room and there is now an established consensus [13]. However, the use of low tidal volumes may precipitate the constitution of pulmonary atelectasis in the poorly ventilated, dependent regions of the lung [14]. Constant low tidal volume ventilation [15] combined with multiple insults from cardiac surgery may engender significant perturbations in lung surfactant homeostasis.

Surfactant, pivotal for maintaining low surface tension and averting atelectasis, undergoes continuous inactivation and/or depletion, necessitating ongoing secretion for its preservation [15]. Meanwhile, animal studies revealed that the diminishment of large aggregate forms of lung surfactant precedes the abnormalities in lung permeability and gas exchange [16]. Mechanical strain on type II pneumocytes, induced notably by large tidal volumes (VTs), represents the strongest stimulus for surfactant release [17–19]. Pattle’s seminal observations nearly six decades ago underscored the significance of yawning or deep breaths in augmenting surfactant recruitment to the alveolar lining film [20]. He postulated that the prevention of deep breaths could precipitate the collapse of alveolar, proposing periodic maximal inflations during mechanical ventilation to forestall such occurrences.

Short-term administration of sighs improves compliance and gas exchange, reducing ventilation heterogeneity and regional lung strain, reversing and preventing atelectasis, and mitigating the production of inflammatory cytokines [21–23]. Recent studies also suggested that sighs seem to be safe when administered to patients with lung injury or trauma [24, 25]. This single-center, randomized study is going to be conducted in patients undergoing scheduled on-pump cardiac surgery to test whether incorporating sigh to perioperative protective ventilation was safe and the efficacy to reduce postoperative hypoxemia and pulmonary complications.

The primary objective is to assess the efficacy of the perioperative sigh breaths in terms of oxygenation during post-extubation period, assessed by time-weighted average SpO_2_/FiO_2_ ratio during the initial post-extubation hour. Key secondary objectives are to assess the severity of respiratory failure by post-extubation day 7 and PPCs by postoperative day 7.

## Methods/design

### Trial design

**Fig. 1:**
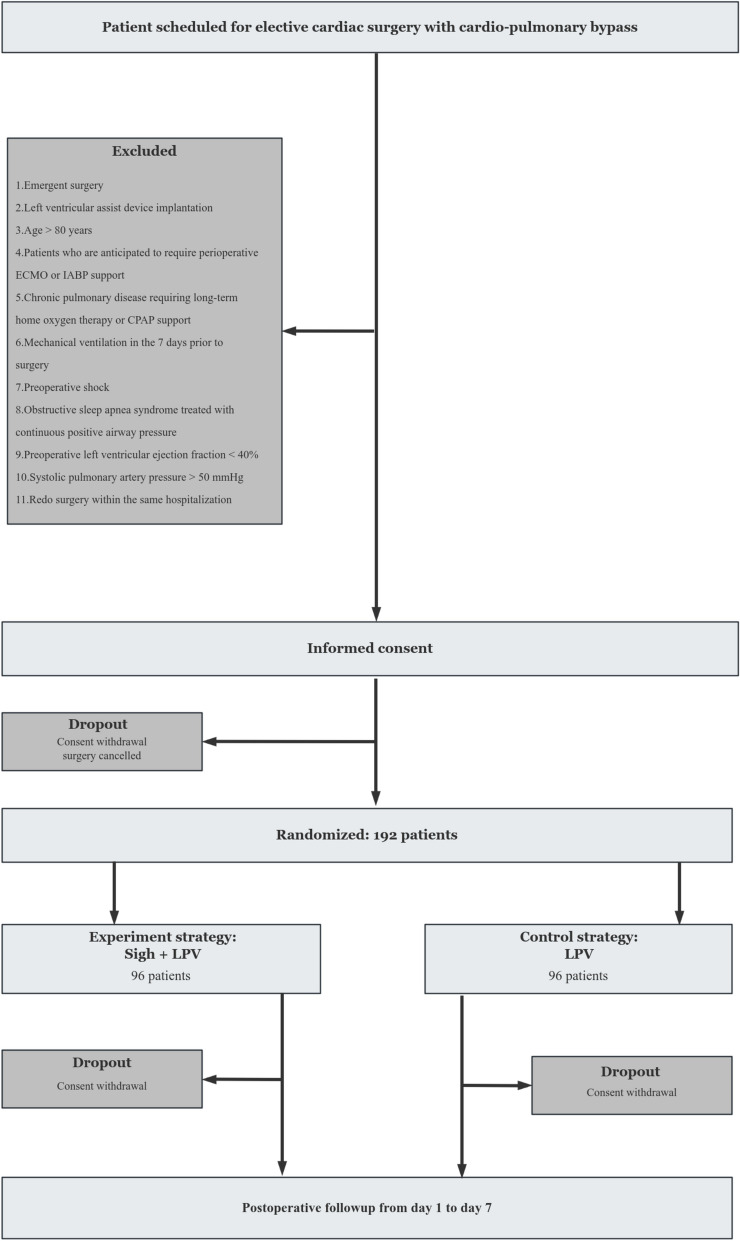
Consolidated Standards of Reporting Trials (CONSORT) diagram for the E-SIGHT trial. ECMO, extracorporeal membrane oxygenation; IABP, intra-aortic balloon pump; CPAP, continuous positive airway pressure; LPV, lung protective ventilation

**Fig. 2:**
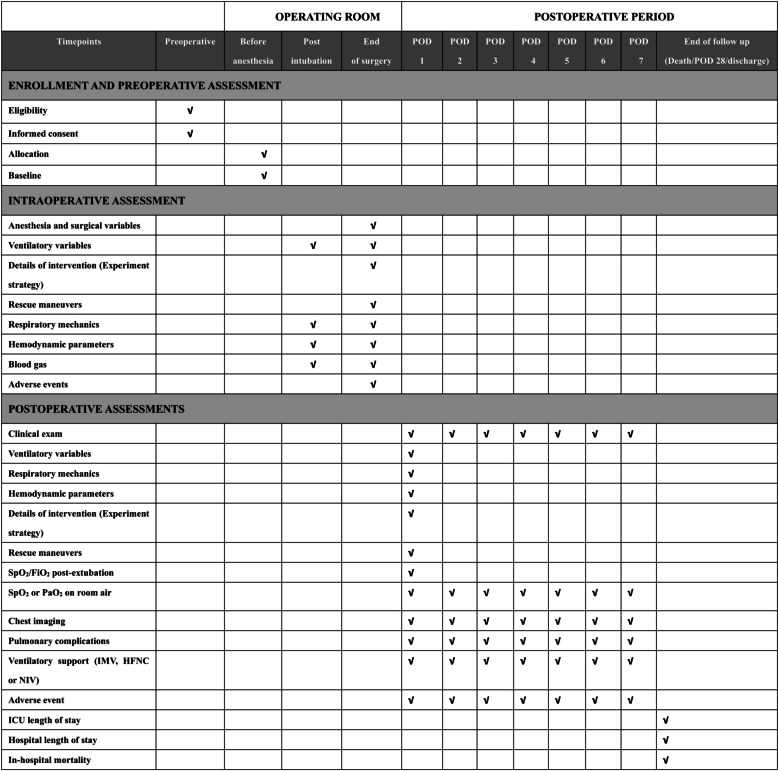
E-SIGHT trial schedule during the study period. *HFNC* high-flow nasal cannula, *ICU* intensive care unit, *IMV* intensive mechanical ventilation, *NIV* non-invasive ventilation, *PaO*_*2*_ arterial pressure in oxygen, *POD* postoperative day, *SpO*_*2*_ pulse oximetry, *FiO*_*2*_ inspired oxygen fraction

This study is an investigator-initiated, prospective, single-center, randomized, controlled, parallel group, clinical trial with a 1:1 allocation. Participants, care providers, and outcomes assessor will be blinded to the group allocation. Two perioperative ventilatory strategies in cardiac surgery with cardiopulmonary bypass are compared: (1) experimental strategy: intermittent sigh breaths plus perioperative lung protective ventilation; (2) control strategy: conventional perioperative lung protective ventilation (Fig. 1). The study will be conducted in adherence to the principles of the World Medical Association’s Declaration of Helsinki and in accordance with the Medical Research Involving Human Subjects Act (WMO). Ethics approval was obtained before starting enrollment and consent will be obtained for each patient following local regulations. The Institutional Review Board of the Zhongda Hospital approved the protocol on 22 Feb 2024 under reference number 2024ZDSYLL037-P01. The trial was registered at www.clinicaltrials.gov with code NCT06248320 in Jan 2024. First patient was enrolled in 25 Feb 2024. The Standard Protocol Items: Recommendation for Interventional Trials (SPIRIT) checklist can be found in Additional file 1 and the SPIRIT figure is included in the main body of the manuscript (Fig. 2).

### Study setting

This clinical trial will be conducted at Zhongda Hospital, a tertiary teaching hospital affiliated to Southeast University, situated in Nanjing, China. This high-volume university hospital performs a minimum of 400 cardiac surgical operations involving CPB annually.

### Eligible criteria

#### Inclusion criteria

Patients are eligible if they are 18 years or older, scheduled for elective cardiac surgery with general anesthesia, conventional CPB, and aortic cross clamp. Signed, informed consent provided by the patient or the legally authorized representatives will be obtained precede the study inclusion.

#### Exclusion criteria

Considering the minimal impact of sigh breaths on individuals with pre-existing hypoxemia resulting from chronic lung diseases, and those with high risk of developing lung dysfunction due to pre-existing severe cardiac impairment or other non-pulmonary risk factors not attributable to perioperative ventilation strategy, these individuals will be excluded from the study.

The exclusion criteria are:

1. Emergent surgery including aortic dissection, cardiac rupture, and active endocarditis surgery
2. Left ventricular assist device implantation
3. Patients who are anticipated to require continuously perioperative circulatory support with extracorporeal membrane oxygenation (ECMO) or intra-aortic balloon pump (IABP)
4. Chronic pulmonary disease requiring long-term home oxygen therapy or continuous positive airway pressure support (CPAP)
5. Receiving invasive mechanical ventilation within 7 days prior to surgery
6. Preoperative shock
7. Obstructive sleep apnea syndrome (OSAS) requiring intermittent non-invasive ventilator support
8. Preoperative left ventricular ejection fraction (LVEF) < 40%
9. Pulmonary arterial systolic pressure > 50 mmHg
10. Redo surgery within the same hospitalization

### Intervention

Mechanical ventilation is performed during general anesthesia and intensive care unit (ICU) on volume-controlled mode. All patients are ventilated with low tidal volumes (6–8 mL/kg of predicted body weight) before and after CPB; ventilation is interrupted during CPB. The predicted body weight is calculated with the formula: 50 + 0.91*(Height in cm − 152.4) in men and 45.5 + 0.91*(height in cm − 152.4) in women. The respiratory rate will be adjusted before and after CPB by the anesthesiologist and/or intensivist to maintain end-tidal CO_2_ partial pressure between 35 and 45 mmHg. The lowest fraction of inspired oxygen (FiO_2_) will be targeted in both groups to maintain SpO_2_ ≥ 96%. The inspiratory to expiratory ratio (I:E) is set at 1:2. Positive end-expiratory pressure (PEEP) is set according to low PEEP/FiO_2_ table for ARDS [26] before and after CPB (Table 1). During sternal sawing, PEEP is temporarily set to 0 cmH_2_O to prevent pleural injury. No additional recruitment maneuvers are performed in both groups.

**Table 1:** Low PEEP/FiO_2_ table

FiO_2_, %	30	40	40	50	50	60	70	70	70	80	90	90	90	100
PEEP, cmH_2_O	5	5	8	8	10	10	10	12	14	14	14	16	18	18–24

The intervention timeframe commences at randomization and concludes at endotracheal extubation, postoperative day 7, or death, whichever occurs first (i.e., during intraoperative and invasive mechanical ventilation in ICU). In the event of reintubation within postoperative day 7, patients will resume the intervention based on their assigned group.

#### Experimental strategy: intermittent sigh breaths plus lung protective ventilation

Sigh breaths are administered by elevating the positive end-expiratory pressure (PEEP), targeting a plateau pressure (Pplat) of 35 cmH_2_O (or 40 cmH_2_O for patients with a body mass index (BMI) > 35 kg/m^2^, considering chest wall compliance would be reduced in those patients). The appropriate delta level of PEEP necessary to achieve the target Pplat is determined manually prior to the initiation of sigh breaths. These sigh breaths will be administered over a 5-s interval once every 6 min, a methodology informed by the findings of Bendixen [27] and SiVent study [24]. Sigh breaths will be administered manually by an anesthesiologist in the operating room, while through ventilators configured to deliver sigh breaths in the ICU, utilizing equipment such as Mindray SV300 or Mindray SV600 or Mindray SV800 or Drager Evita Infinity V500, depending on availability.

Sigh breaths are administered at predetermined intervals throughout the entire duration of invasive mechanical ventilation:

1. From endotracheal intubation to the surgical opening of the chest cavity
2. From the surgical closure of the chest cavity to exiting the operating room
3. From ICU admission to the start of the spontaneous breathing trial

Patient needs not continue to receive sigh breaths during the time out of operating room and ICU (i.e., transported to CT, transported from operation department to ICU. Sigh breaths should be restarted on their return).

Subsequent sigh breaths will be suspended if the systolic arterial pressure drops below 90 mmHg, despite adequate administration of fluids and/or vasoactive drugs. Sigh breaths will resume once the systolic arterial pressure returns to ≥ 95 mmHg and remains stable for at least 5 min. Records of sigh interruption will be documented and analyzed (Table 2).

**Table 2:** Perioperative ventilatory protocol in each of the two treatment arms

	**Experimental strategy**	**Control strategy**
From endotracheal intubation to chest cavity opening	LPV + sigh breaths	Lung protective ventilation (LPV) • Tidal volume 6–8 mL/kg PBW • PEEP: ARDS low PEEP-FiO_2_ table • RR ETCO_2_ 35–45 mmHg • FiO_2_ lowest to maintain SpO_2_ ≥ 96% • I:E ratio 1:2
Lung recruitment maneuvers	No	No
During chest cavity opening	Lung protective ventilation	Lung protective ventilation
During CPB	Ventilation interrupted	Ventilation interrupted
Ventilation after chest cavity closure (including in ICU)	LPV + sigh breaths	LPV

*LPV* lung protective ventilation, *ICU* intensive care unit, *CPB* cardiopulmonary bypass, *FiO*_*2*_ inspired oxygen fraction, *I:E* inspiratory time to expiratory time ratio, *PEEP* positive end-expiratory pressure, *PBW* predicted body weight, *RR* respiratory rate, *SpO*_*2*_ pulse oximetry, *ETCO*_*2*_ end-tidal CO_2_

#### Control strategy: lung protective ventilation

Patients are ventilated with low tidal volumes (6–8 mL/kg of predicted body weight) before and after CPB; ventilation is interrupted during CPB. PEEP is set according to low PEEP/FiO_2_ table for ARDS. Ventilation is interrupted during CPB (Table 2).

#### Rescue therapy

In both groups, unplanned recruitment maneuvers are permitted as rescue therapy in case of hypoxemia during the intervention period. Hypoxemia is determined as SpO_2_ ≤ 92% despite FiO_2_ of 80%.

### Standard of care

#### Surgery

The type of drugs used for the anesthesia, the management of the CPB, and fluid and transfusion strategies are implemented according to local protocol.

#### Extubation protocol

Patients will be excluded from the protocol if one of the following criteria is met:

1. Postoperative continuous drainage exceeding 100 mL per hour for 3 consecutive hours.
2. Left ventricular ejection fraction less than 30% or signs suggesting persistent deteriorating heart failure.
3. Presence of escalating pericardial effusion or evident manifestations of obstructive shock.
4. Perturbations in internal milieu: pH below 7.35 or above 7.45, serum potassium less than 3.0 mmol/L or greater than 5.5 mmol/L; blood lactate concentration ≥ 6 mmol/L.
5. Hourly urine output less than 0.5 mL/kg/h.
6. Difficulty in endotracheal intubation during anesthesia.
7. Persistent hypothermia despite aggressive rewarming measures, with temperatures below 36 °C.
8. Newly onset arrhythmias: heart rate below 40 beats/min or above 140 beats/min; ventricular rhythm disturbances; atrial fibrillation accompanied by hemodynamic instability.

After excluding the aforementioned conditions, the patient will enter a rapid extubation protocol. The tidal volume will remain unchanged, while adjusting the positive end-expiratory pressure (PEEP) to 5 cmH_2_O. Sedative medications will be discontinued, and analgesic medication dosages will be adjusted personally. During this period, close monitoring of the patient’s oxygenation, blood pressure, and other vital signs will be conducted. After 1–2 h, depending on the patient’s arousal status, readiness for a spontaneous breathing trial (SBT) will be assessed. Patients meeting the following criteria will undergo an SBT:

1. Pulse oximetry saturation ≥ 92% given inspired oxygen fraction (FiO_2_) ≤ 40%.
2. Alertness, able to follow commands, without agitation (Richmond Agitation-Sedation Scale [RASS] score of − 2 to 0).
3. Hemodynamically stable (norepinephrine dose ≤ 0.1 µg/kg/min or equivalent doses of other vasopressor agents; or gradual tapering of vasopressor medications over the past 4 h).
4. No potential short-term need for controlled ventilation due to other reasons.

During the 30-min spontaneous breathing trial (SBT) conducted in pressure support ventilation (PSV) mode with PEEP set at 5 cmH_2_O and PSV between 5 and 10 cmH_2_O, if any of the following conditions are met at the end of the 30-min period, the patient will fail the SBT:

1. Respiratory rate > 35 breaths per minute or significant accessory muscle involvement.
2. Oxygen saturation (SpO_2_) < 92%.
3. Heart rate > 140 beats per minute or an increase in heart rate > 20%, or newly onset arrhythmias.
4. Systolic blood pressure > 180 mmHg or < 90 mmHg.
5. Patient reports significant respiratory distress.
6. Evidence of active myocardial ischemia (dynamic ST-segment changes on electrocardiogram monitoring).
7. Significant alteration in consciousness (Glasgow Coma Scale [GCS] score < 13, Richmond Agitation-Sedation Scale [RASS] score <  − 3), weak cough effort, or inadequate airway clearance capability.

Patients who fail the SBT will be reverted to controlled ventilation or pressure support ventilation mode as deemed appropriate. A subsequent SBT screening and assessment will be conducted if the symptoms listed above are effectively relieved, with ventilation continued according to the predefined group allocation.

#### Post-extubation ventilatory support

After extubation, routine oxygen therapy is typically administered via nasal cannula or face mask. For patients receiving face mask oxygen therapy at a flow rate of ≥ 5 L/min and failing to achieve a pulse oximetry level greater than 92% or respiratory rate ≤ 25 breaths/min will be considered for escalation to high-flow oxygen therapy (HFNO) or non-invasive ventilation (NIV), specific option depends on the clinical judgment of the attending physician, and no priorities will be predefined [28].

##### High-flow nasal oxygen (HFNO) therapy

Initial parameters for HFNO therapy include a flow rate of 30 L/min and an oxygen concentration of 100%. Subsequently, oxygen concentration will be titrated to maintain pulse oximetry within the range of 92–98%, and adjust the flow rate to the maximum tolerated by the patient, all maneuvers will completed within the first 10 min after HFNO therapy initiation. Subsequent adjustments in oxygen concentration and flow rate are made based on the target pulse oximetry (SpO_2_ 92–98%) and personal tolerance. If the patient meets the criteria of FiO_2_ less than 40% and respiratory rate < 25 breaths/min after high-flow therapy, an HFNO weaning trial will be conducted.

Maintaining the oxygen concentration and reduce the flow rate to 10 L/min, and maintain for 10 min. If the patient maintains a pulse oximetry between 92 and 98% and a respiratory rate of ≤ 25 breaths/min during the trial, HFNO can be discontinued, and conventional nasal cannula or face mask oxygen therapy can be initiated. If the patient does not pass the HFNO weaning trial, HFNO therapy will continue.

For patients who fail the HFNO weaning trial for the first time, if they subsequently meet the following criteria during HFNO therapy: FiO_2_ less than 40%, flow rate less than 30 L/min, and respiratory rate < 25 breaths/min, consideration may be given to discontinuing HFNO therapy and switching to regular nasal cannula or face mask oxygen therapy after passing the HFNO weaning trial. If oxygen saturation falls below 92% or respiratory rate exceeds 25 breaths/min during subsequent treatment, HFNO therapy can be resumed.

##### Non-invasive ventilation (NIV)

Initial parameters for NIV include an inspiratory positive airway pressure (IPAP) of 8 cmH_2_O, expiratory positive airway pressure (EPAP) of 5 cmH_2_O, and FiO_2_ of 100%. Subsequently, IPAP is adjusted within the range of 5–15 cmH_2_O to achieve a tidal volume of 6–8 mL/kg and a respiratory rate < 25 breaths/min. FiO_2_ is adjusted within the range of ≤ 60%, and EPAP within the range of 5–10 cmH_2_O to maintain SpO_2_ between 92 and 98%. Once oxygenation and respiratory distress symptoms improved, intermittent use of NIV will be considered, and NIV can be discontinued if applied for less than 4 h daily.

#### Reasons for reintubation

Reintubation indications include emergent anesthesia intubation for postoperative complications such as significant bleeding or cardiac tamponade requiring urgent reoperation, altered consciousness (Glasgow Coma Scale [GCS] score < 12), cardiac arrest or malignant arrhythmias, severe hemodynamic instability (norepinephrine dose > 0.1 µg/kg/min or equivalent doses of other vasopressor agents), and deteriorating respiratory failure (meeting at least two of the following criteria): unresolved hypoxemia (PaO_2_ < 60 mmHg or pulse oximetry persistently below 90% despite high-flow nasal oxygen [HFNO] at a flow rate exceeding 30 L/min and 100% oxygen concentration), respiratory acidosis (pH < 7.30, PaCO_2_ > 50 mmHg), respiratory rate > 30 breaths/min, inability to effectively clear airway secretions.

## Standard procedures

### Screening and inclusions

Patients are screened for inclusion and exclusion criteria during the preoperative visit by anesthesiologist. Patients who meet all inclusion criteria and do not meet any exclusion criteria will be included in the study after providing written, signed informed consent.

### Randomization and allocation

The computer-generated randomized lists were drawn up by an independent biostatistics expert before the beginning of the study, using a permuted block design. Biostatistics will employ the SAS 9.4 statistical software PLAN procedure statement on a computer, specifying a seed, to generate randomized treatment assignments for sample subjects. The allocation will be implemented automatically in the electronic case report form (REDCap 10.6.9—© 2024 Vanderbilt University, USA). Allocation concealment will be ensured, as the anesthesiologist will assign the patient to the study group only upon the patient’s arrival in the operating room, ensuring that withdrawal of informed consent has not occurred, and after all baseline measurements will be completed.

### Masking

Trial participants will be blinded to group assignment. Assessments regarding study outcomes will be conducted by an assessor blind to treatment allocation, ensured by concealment of group allocation in the REDCap system to outcome assessor. The assessor will go through a profound assessment training program. Due to the necessity of data collection during intervention, investigators occupied for data collection could not be blinded to allocation, but are strongly inculcated not to disclose the allocation status of the participant at the follow-up assessments. Data analysts can analyze data only after study completion without having access to information about the allocation.

Unblinding to study participants will happen only after the study completion and at participants’ request.

### End of follow-up

Patients enrolled will be followed up until postoperative day 28, hospital discharge, or death, whichever comes first.

### Study endpoints

Given the high sensitivity and specificity of SpO_2_/FiO_2_ in diagnosing acute lung injury without the need for repeated blood sampling [29], we consider it to be a suitable alternative to PaO_2_/FiO_2_. The primary endpoint is time-weighted average SpO_2_/FiO_2_ ratio during the initial post-extubation hour. We first calculated the SpO_2_/FiO_2_ ratio every 15 min during the initial post-extubation hour, and then averaged all SpO_2_/FiO_2_ ratios weighted by measurement interval. Adequate waveform and proper oximeter placement will be confirmed during the initial post-extubation hour. Oxygen flow will be adjusted to maintain pulse oximetry between 92 and 97% during the initial post-extubation hour and ensuring stability for at least 5 min before measurement at each time point. The relationship between oxygen flow and estimated inspired oxygen concentration through different oxygen delivery devices is listed in the Additional file 2.

The secondary endpoints include:

1. Respiratory failure by post-extubation day 7
  No respiratory failure: SpO_2_ ≥ 90% after breathing ambient air for 10 min (excluding hypoventilation)
  Mild respiratory failure: SpO2 < 90% or PaO2 < 60 mmHg after breathing ambient air for 10 min (excluding hypoventilation) and corrected with 1–3 L/min with a nasal cannula
  Moderate respiratory failure: SpO2 < 90% or PaO2 < 60 mmHg despite a 3 L/min oxygen supply with a nasal cannula (excluding hypoventilation) and corrected with an oxygen supply from 4 to 10 L/min with a face mask
  Severe respiratory failure: SpO2 < 90% or PaO2 < 60 mmHg despite a 10 L/min oxygen supply with a face mask (excluding hypoventilation) and corrected with an oxygen supply > 10 L/min with a high-flow face mask or with non-invasive ventilation or with high-flow nasal oxygen therapy or with invasive mechanical ventilation
2. Severity of postoperative pulmonary complications by postoperative day 7, scored on an ordinal scale ranging from 0 to 5, using a modified definition of pulmonary complications [10]
  Grade 0: No symptoms or signals
  Grade 1: One of the following: dry cough, abnormal lung findings and temperature 37.5 °C or higher with normal chest radiograph, or dyspnea without other documented cause
  Grade 2: Two of the following: productive cough, bronchospasm, hypoxemia (SpO2 90%) at room air, atelectasis with gross radiological confirmation (concordance of 2 independent experts) plus either temperature higher than 37.5 °C, or abnormal lung findings, hypercarbia (PaCO2 > 50 mmHg) requiring treatment
  Grade 3: One of the following: pleural effusion resulting in thoracentesis, pneumonia, pneumothorax, extended noninvasive ventilation or high-flow nasal cannula, or reintubation lasting less than 48 h
  Grade 4: Reintubation or invasive mechanical ventilation for 48 h or more
  Grade 5: Death before hospital discharge
3. Invasive mechanical ventilation (IMV) days in ICU by postoperative day 7
  Duration of invasive mechanical ventilation by postoperative day 7, initiated from ICU
  admission.
4. Use of high-flow nasal cannula or non-invasive ventilation by postoperative day 7.
5. Use of new invasive mechanical ventilation by postoperative day 7.
6. No ventilatory support days by postoperative day 7.
  No ventilatory support days are defined as days without invasive mechanical ventilation,
  high-flow nasal cannula, and non-invasive ventilation.
7. In-hospital mortality
  Death occurred during hospital stay, time frame from the day of surgery up to hospital
  discharge or death, maximum censoring at day 28 after surgery.
8. ICU length of stay
  Days since surgery until ICU discharge. The censoring will be performed at day 28 after
  surgery. Patients dying before leaving the ICU were censored as not discharged from ICU at
  day 28 after surgery.
9. Length of hospital stay
  Days since surgery until hospital discharge. The censoring was performed at 28 days. Patients
  dying before leaving the hospital were censored as not discharged from hospital at day 28.
  Adverse events by postoperative day 7:
  Use of high-dose vasopressors (> 1 µg/kg/min of norepinephrine)
  Postoperative barotrauma
  Defined as the presence of radiological evidence suggesting pneumothorax, mediastinal emphysema, new-onset pulmonary emphysema, or subcutaneous emphysema.
  Reoperation before the 12th postoperative hour

### Data collection

Study data are managed with a password-protected electronic case report form (REDCap 10.6.9—Copyright 2024 Vanderbilt University, USA). Patient data will be anonymous and coded according to a number. The eCRF includes tools to promote data quality, such as range checks for data values. Data monitoring will be performed by means of queries on the database done by statisticians and analyzed to identify abnormalities and inconsistencies.

All participants will be informed that they will be followed up until postoperative day 28, hospital discharge, or death, whichever occurs first, to ensure the completion of surveys upon consent. Participants choosing to withdraw consent to the study will be encouraged to continue with the follow-up of the trial for the evaluation of efficacy and safety. The reasons and circumstances for discontinuing the study will be recorded.

#### Baseline data

The following baseline data are collected after the patient’s inclusion: gender, age, height, weight, BMI, smoking status, history of COPD or asthma with chronic inhalation therapy, American Society of Anesthesiologists (ASA) score, Euroscore II, lower respiratory tract infection in the past 3 months, abnormal preoperative chest imaging, cardiovascular status (diabetes mellitus, arterial hypertension, dyslipidemia, liver disease, previous acute myocardial infarction, history of cardiac surgery, left ventricular ejection fraction, echocardiographic right ventricular distention defined by a right ventricle/left ventricle ratio > 1, brain natriuretic peptide level, cardiac troponin I level), and status of preoperative creatininemia > 3 mg/dL.

#### Intraoperative variables

During the surgery, the anesthesiologist in charge of the patient will record the following variables: type of surgery (coronary artery bypass graft, valve surgery, aortic surgery, mixed surgery, none of the above), site of vascular graft harvesting (great saphenous vein, mammary artery, radial artery), number of vessels for coronary arterial bypass, CPB duration, aortic cross clamp duration, surgical duration, intraoperative fluid volume (crystalloid and colloid), fluid balance during surgery, use of blood transfusions (include red cell, platelets, or plasma), use of inotropic agents and vasopressors, use of rescue therapy. Gas exchange parameters (arterial blood partial pressure of carbon dioxide, arterial blood partial pressure of oxygen, PaO_2_/FiO_2_), mechanical ventilation parameters (tidal volume, respiratory rate, positive end-expiratory pressure, inspired oxygen fraction, minute ventilation), and respiratory mechanics (peak airway pressure, plateau pressure, driving pressure, respiratory system compliance) will be recorded within 10 min after endotracheal intubation and within 10 min after the conclusion of the surgery.

For patients allocated to the experimental strategy, further details of the experimental intervention will be documented as follows: the total duration of sigh breaths during the intraoperative period. Hemodynamic variables, including heart rate, systolic arterial pressure, diastolic arterial pressure, and mean arterial pressure, will be assessed during the first and second intraoperative sigh breath. Any interruptions in sigh breaths during the intraoperative period will also be documented.

#### Postoperative variables

Gas exchange parameters, mechanical ventilation parameters, and respiratory mechanics will be recorded within 10 min, 2 h, and 4 h after ICU admission. Use of rescue therapy will also be recorded during invasive mechanical ventilation in postoperative days. For patients allocated to the experimental strategy, total duration of sigh breaths during the postoperative period, hemodynamic variables, and any interruption of sigh breaths will be documented.

For outcome assessments, SpO_2_/FiO_2_ will be assessed during the initial post-extubation hour, measured every 15 min. Then, patients are visited, twice a day, every postoperative day until postoperative day 7, in order to assess the presence of PPCs or other secondary endpoints. Hospital mortality, length of hospital stay, and length of ICU stay will be assessed upon hospital discharge, death, or postoperative day 28, whichever occurred first. The need for supplemental oxygen will be evaluated at each visit by measuring SpO_2_ and/or PaO_2_ after 10 min of breathing room air. Lung auscultation and daily symptoms of cough and expectoration will also be assessed at each visit. A daily chest X-ray will be prescribed during ICU stay. In the surgical ward, a chest X-ray will be prescribed at the discretion of the attending physician. A chest CT scan will be conducted between the third and fifth postoperative day.

### Sample size and power

The study determined a sample size of 192 patients (96 per group) with a 5% dropout rate and a *P* value of 0.05 to achieve 90% power in detecting a 10% increase in time-weighted average SpO_2_/FiO_2_ during the initial post-extubation hour in the experiment strategy group, compared to the control group. The control group’s mean time-weighted average SpO_2_/FiO_2_ was estimated to be 294 with a standard deviation of 60, based on previous research [30], and the 10% difference between groups was determined from previous work showing that a decrease of 10% from baseline in PaO_2_/FiO_2_ was clinically meaningful in lung injury induced by ventilatory strategies [31–33]. A dropout rate of 5% was deemed acceptable for informed consent withdrawals post-enrollment.

### Statistical analysis

The data will be analyzed using R (version 4.3.3). The primary analysis will be carried out according to the intention-to-treat principle. The full analysis population (including all subjects who will be randomized and will be at least evaluated at baseline) will be used in the primary analysis. No interim analysis is planned. In case of missing data, the reason and mechanism for missing data will be explored. For missing data greater than 20%, multiple imputation may be considered as a sensitivity analysis to evaluate the treatment effect and associated standard error as appropriate.

Categorical variables are reported as number and percentage. Normally distributed variables are reported as mean and SD, non-normally distributed as median and IQR. Normal distribution of data will be assessed with the Kolmogorov–Smirnov test or the Shapiro–Wilk test. To compare continuous variables between experimental strategy and control strategy groups, Student’s *t*-test or Mann–Whitney *U* test will be used, as appropriate. Categorical variables will be compared using the Fisher exact test or likelihood ratio tests. The baseline and intraoperative parameters will be described per group (“experimental strategy” and “control strategy”) in accordance with the Consolidated Standards of Reporting Trials (CONSORT) guidelines.

The time-weighted average SpO_2_/FiO_2_ ratio will be compared as continuous variable. And treatment effects of primary outcome will also be analyzed according to the following subgroups: (1) age; (2) duration of cardiopulmonary bypass; (3) preoperative pathological chest image; (4) red cell transfusion during surgery. For secondary outcomes, the scores of pulmonary complications and severity of respiratory failure will be analyzed through Mann–Whitney *U* tests and multivariable ordinal logistic regression by estimating the common odds ratio for a shift in the direction of a better outcome on the modified scale. The proportion of other secondary endpoints (use of high-flow nasal cannula or non-invasive ventilation, use of new invasive mechanical ventilation, in-hospital mortality, adverse events) will be compared between the groups. Non-ventilatory support days and invasive mechanical ventilation days by postoperative day 7 will be compared between groups as continuous variables. Multiple comparison corrections will be performed for non-independent outcomes. The duration of ICU and hospital stay was compared using Kaplan–Meier curves and log-rank tests. The censoring was performed at postoperative day 28, and the time to event was the time from surgery end to ICU and hospital discharge. Patients who died before leaving the ICU or hospital were censored as nondischarged at day 28. A 2-sided *P* value < 0.05 was considered statistically significant.

### Oversight and monitoring

No data monitoring committee (DMC) will be established in this trial as our investigator team could closely monitor participants’ safety and data integrity. Trial steering committee comprises two professors of intensive care medicine (JYX and JFX), a professor of cardiac surgery (HLC), a professor of anesthesiology (JS), and two independent statisticians. The trial steering committee convenes quarterly throughout the trial period to review trial conduct. This committee will validate the protocol, oversee the study, and have the authority to make decisions regarding its smooth running and publication strategy. And the Ethics Committee of Zhongda Hospital meets semi-annually or as needed to evaluate trial’s ethical conduct and the ethics committee has the authority to recommend modifications to the trial or its termination if significant concerns arise.

Any unforeseen medical events occurring in participants during the intervention period will be categorized as adverse events (AEs). Serious adverse events (SAEs) are defined as adverse events, including death, life-threatening situations, permanent or severe disability or loss of function, and the need for prolonged hospitalization, that occur following the trial treatment. All AEs will be meticulously recorded in the case report form (CRF), and SAEs will be promptly reported to the principal investigators. Upon identification of an AE, investigators will thoroughly assess the association between the intervention and the AE and determine whether to discontinue the intervention. All serious, unexpected, and study-related adverse events will be reported to the Ethics Committee of Zhongda Hospital within 15 calendar days. Patients who discontinue the intervention due to an AE will continue to be followed up. All patients in this study will be hospitalized in Zhongda Hospital. Accordingly, emergency care will be readily available at all times. The cost of treatment for all study-related adverse events will be covered by the trial initiator. The ancillary and post-trial care will be provided by a medical team, consist of cardiac surgeons, nurses, rehabilitation therapists, and intensivists, after the trial is completed.

Any modifications to the study protocol will necessitate synchronous protocol amendments, which will be promptly submitted for approval to the Ethics Committee/Institutional Review Board. These changes will only be implemented after approval by the ethical committee. Once approved, ClinicalTrials.gov will be promptly updated regarding any significant changes. If required, protocol training for the amendments will be provided by the study team.

## Discussion

The E-SIGHT trial is the first single-center, randomized controlled trial designed to evaluate the potential benefits of augmenting lung protective ventilation (LPV) with sigh breaths during the perioperative period in patients scheduled for cardiac surgery. This study aims to ascertain the feasibility and preliminary efficacy of this intervention in comparison to the conventional LPV approach. Should the feasibility be established, the E-SIGHT trial will lay the groundwork for a subsequent, more extensive clinical trial to definitively determine the efficacy of the sigh + LPV strategy in reducing postoperative pulmonary complications (PPCs). Furthermore, this research endeavors to introduce and assess a novel perioperative ventilation approach to mitigate the risk of postoperative hypoxemia and PPCs in patients undergoing cardiac surgery, ultimately enhancing their recovery in the postoperative period.

In the experimental arm, we have chosen a multimodal approach, from intubation to extubation, in order to reflect the prophylactic use nature of sighs, counteract with the risk of atelectasis which persists during the entire mechanical ventilation period. Sigh will not be administered during the opening of chest cavity giving the potential impact on the surgical procedure. The plateau change resulting from increasing PEEP producing a Pplat of 35 or 40 cmH_2_O was selected for two reasons. First, it produces an end-inspiratory lung volume that approximates total lung capacity in patients with normal chest wall and lung compliances, thereby facilitating secretion of surfactant. Second, a Pplat of 40 cmH_2_O has been utilized in numerous short-term studies of recruitment maneuvers in patients with ARDS without any evidence that it caused barotrauma or volutrauma [24]. In those patients with BMIs exceeding 35, the volume of the sigh breath will be determined on the basis of a Pplat of 40 cmH_2_O given the decrease in chest wall compliance that will be present. We did not choose an individualized PEEP titration protocol because of the absence of a validated reference titration protocol and because of the high risk of hemodynamic intolerance and barotrauma [34]. In both groups, PEEP level is selected according to low FiO_2_-PEEP table adopted from ARDS studies, its pragmatic design limited burden on daily activities and is moderate in comparison with previous studies in cardiac surgery [26]. During CPB, apnea was performed as no firm evidences of benefits of ventilation maintenance during CPB until nowadays [12, 35].

The primary outcome was the time-weighted average SpO_2_/FiO_2_ ratio during the initial post-extubation hour. Given the high sensitivity and specificity of SpO_2_/FiO_2_ in diagnosing acute lung injury without the need for repeated blood sampling [29], we consider it to be a suitable alternative to PaO_2_/FiO_2_. This is a very pragmatic endpoint, and previous performance in postoperative patients has proved good external validity [36]. To complementary for the primary endpoint, we evaluated long-term respiratory failure and postoperative pulmonary complications in the secondary outcomes, and the specific definitions allow for comparisons with previous studies [10, 12]. The need for non-invasive ventilation or high-flow nasal oxygen therapy has also been included in the secondary outcomes and standard procedures of postoperative ventilatory support were provided in the protocol. Finally, we will evaluate the invasive mechanical ventilation days during ICU stay, non-ventilatory support days, and endotracheal reintubation rate in postoperative day 7. Evaluating different outcomes with tight relationship with postoperative pulmonary function recovery will give a relatively comprehensive point of view on the impact of sigh approach.

One important limitation of the E-SIGHT trial is that double-blinding is not possible due to the nature of intervention. However, the outcome assessor will be blinded to the randomization arms, ensured the reliability of the study results. Second, in the absence of conclusive data on sigh “minimum effective dose,” we chose a rate of 5 s per 6 min because this frequency is considered to be close to the physiological state of a normal person [27]. Finally, we did not plan any biomolecular analysis to verify the impact of addition of sigh on lung protection for budget reasons; in any case, we will collect a huge amount of physiological data during the whole study period that will allow reconstruction on mechanistic effects underlying the clinical benefits.

In conclusion, E-SIGHT is a single-center randomized controlled trial to test the feasibility of long-term addition of sigh to lung protective ventilation. Its results could provide a ready-to-use treatment enhancing perioperative lung protection. Moreover, E-SIGHT will be the basis for planning a future larger trial investigating the use of sigh as a strategy to improve hard clinical outcomes in patients undergoing cardiac surgery through enhanced lung protection.

## Trial status

The E-SIGHT trial is currently recruiting patients. The protocol was approved by the Institutional Review Board on 22 February 2024. Current protocol version number was version 2, 10th Feb 2024. The first patient was enrolled on 25 February 2024. Recruitment is expected to be completed in October 2024.

## Supplementary Information

Additional file 1: SPIRIT 2013 checklist: recommended items to address in a clinical trial protocol and related documents.

Additional file 2: eTable 1 Estimation of inspired oxygen fraction through different oxygen devices.

## Data Availability

Any data collected in this study can be obtained from the corresponding author with a reasonable request.

## Abbreviations

CPB: Cardiopulmonary bypass
LPV: Lung protective ventilation
PEEP: Positive end-expiratory pressure
ARDS: Acute respiratory distress syndrome
PPCs: Postoperative pulmonary complications
ECMO: Extracorporeal membrane oxygenation
IABP: Intra-aortic balloon pump
BMI: Body mass index
VTs: Tidal volumes
SpO_2_: Pulse arterial oxygen saturation
FiO_2_: Fraction of inspiration oxygen
I:E: Inspiratory to expiratory ratio
Pplat: Plateau pressure
IPAP: Inspiratory positive airway pressure
EPAP: Expiratory positive airway pressure
VILI: Ventilator-induced lung injury
HFNO: High-flow nasal oxygen
CPAP: Continuous positive airway pressure
NIV: Non-invasive ventilation
SBT: Spontaneous breathing trial
OSAS: Obstructive sleep apnea syndrome
LVEF: Left ventricular ejection fraction
RASS: Richmond Agitation-Sedation Scale
GCS: Glasgow Coma Scale
CONSORT: Consolidated Standards of Reporting Trials
SPIRIT: Standard Protocol Items: Recommendation for Interventional Trials
