# Supplementary material for: Effect of perioperative sigh ventilation on postoperative hypoxemia and pulmonary complications after on-pump cardiac surgery (E-SIGHT): study protocol for a randomized controlled trial
Source: Trials. 2024 Sep 4;25:585. doi: 10.1186/s13063-024-08416-y (PMC11373100; doi:10.1186/s13063-024-08416-y)
Supplement: Supplementary file 2 — Additional file 2: eTable 1 Estimation of inspired oxygen fraction through different oxygen devices. [file 13063_2024_8416_MOESM2_ESM.docx]

eTable 1. Estimation of inspired oxygen fraction through different oxygen devices.

| Oxygen device | O_2_ flow (L/min) | Estimated FiO_2_ (%) |
| --- | --- | --- |
| Nasal cannula | 1 | 24 |
|  | 2 | 28 |
|  | 3 | 32 |
|  | 4 | 36 |
|  | 5 | 40 |
|  | 6 | 44 |
| Face mask | 5 | 40 |
|  | 6-7 | 50 |
|  | 7-8 | 60 |
